# Damage limitation

**DOI:** 10.7554/eLife.17394

**Published:** 2016-05-23

**Authors:** Ivano Amelio, Gerry Melino

**Affiliations:** 1 Medical Research Council Toxicology Unit, Leicester University, Leicester, United Kingdomia119@leicester.ac.uk; 1 Medical Research Council Toxicology Unit, Leicester University, Leicester, United Kingdomgm89@leicester.ac.uk; 2 Department of Experimental Medicine and Surgery, University of Rome Tor Vergata, Rome, Italy

**Keywords:** DNA damage, kinetically trapped state, p63, quality control, spring-loaded activation, oocytes, p53 family proteins, *E. coli*, Mouse

## Abstract

A spring-loaded mechanism can explain the activation process for a protein that has a crucial role in maintaining the genomic integrity of immature eggs cells

**Related research article** Coutandin D, Osterburg C, Srivastav RK, Sumyk M, Kehrloesser S, Gebel J, Tuppi M, Hannewald J, Schäfer B, Salah E, Mathea S, Müller-Kuller U, Doutch J, Grez M, Knapp S, Dötsch V. 2016. Quality control in oocytes by p63 is based on a spring-loaded activation mechanism on the molecular and cellular level. *eLife* **5**:e13909. doi: 10.7554/eLife.13909

**Image** TAp63α tetramers (gold) trigger cell death in oocytes if DNA damage is not repaired

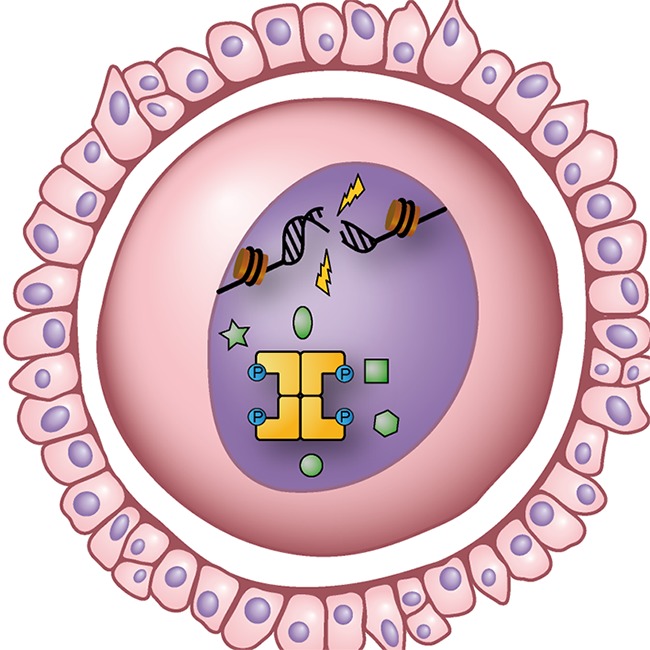

Members of the p53 family of transcription factors help to prevent mutations building up in DNA and therefore act to maintain the integrity of animal genomes. TAp63α is the oldest protein in this family and its role is to stop oocytes (immature egg cells) dividing if their DNA has been damaged. And if any DNA damage is not repaired, TAp63α triggers the death of the cell (Levine et al., 2011).

Oocytes form as embryos develop, but many die before birth. After birth, the remaining oocytes produce high levels of TAp63α and this makes them extremely vulnerable to DNA damage: indeed, relatively low amounts of DNA damage (fewer than 10 double-strand breaks) can kill an oocyte. However, this vulnerability ensures that only those oocytes with stable genomes will survive to the point where they could be fertilized during reproduction (Suh et al., 2006).

Individual TAp63α units usually pair up to form dimers that are not active, but when DNA damage is detected, two dimers link up to form a tetramer that is active (Deutsch et al., 2011). The fact that TAp63α is usually in an inactive state prevents unintended cell death and thus preserves the limited reservoir of oocytes, which cannot be replaced after an animal is born. Now, in eLife, Volker Dötsch at Goethe University in Frankfurt and colleagues – including Daniel Coutandin and Christian Osterburg as joint first authors – report the result of detailed biophysical experiments on these dimers and tetramers (Coutandin et al., 2016).

Two of the domains in TAp63α – the C-terminal transactivation inhibitory (TI) domain and the N-terminal transactivation (TA) domain – are known to be involved in stabilizing the inactive dimeric form of the TAp63α. Coutandin et al. used alanine scanning and small angle X-ray scattering techniques to reveal that a structure called a β-sheet blocks the formation of the tetramer by covering the regions of the TAp62α units that interact to form the tetramer. This β-sheet is formed when a part of the TA domain in one unit interacts with the TI domain in the other unit, and vice versa.

Phosphorylation is the process that triggers the transition from the dimeric state to the active tetramer. In oocytes, several kinase enzymes are known to activate the phosphorylation of TAp63 when DNA is damaged (Gonfloni et al., 2009; Bolcun-Filas et al., 2014). However, the fact that the activation process is not reversible led many researchers to question if phosphorylation really does change the biophysical properties of the TAp63α tetramer to make it more stable than the dephosphorylated form.

Coutandin et al. – who are based at Goethe University, Merck, Oxford, Georg-Speyer Haus and the Rutherford Appleton Laboratory – propose that a "spring-loaded mechanism" can explain the biophysical basis of TAp63 activation (Figure 1). In this model, phosphorylation acts as the trigger that opens up the TAp63 dimers and allows them to form tetramers. The activation process is irreversible because the tetramer is more stable than the dimer: in other words, the energy of the tetramer state is lower than the energy of the dimer state.

**Figure 1.**
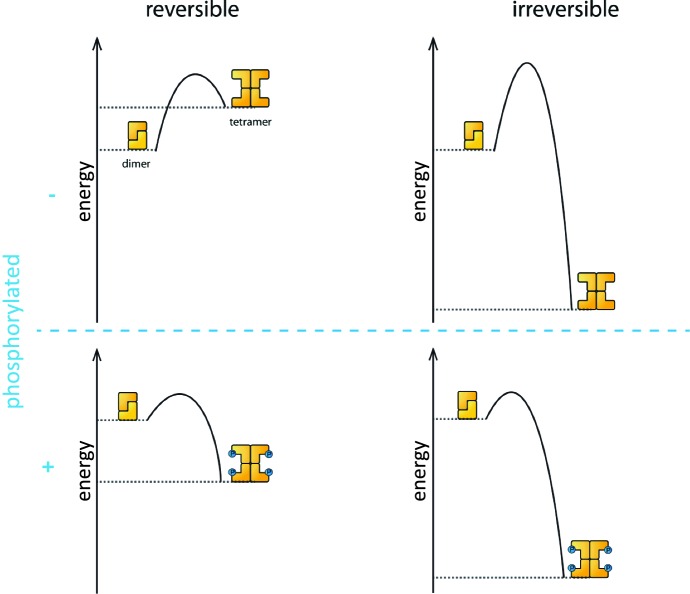
Activation of TAp63α. The process that causes inactive TAp63α dimers to become active TAp63α tetramers is triggered by phosphorylation (blue dots; blue plus sign). However, the precise role played by phosphorylation was not fully understood. The simplest assumption would be that the dimer is more stable than the tetramer (that is, the energy of the dimer state is lower than the energy of the tetramer state; top left), and that phosphorylation makes the tetramer more stable (bottom left). Under this assumption the activation process would be reversible and dephosphorylation (blue minus sign) would return the system to its original state (top left). However, experiments showed that the activation process was irreversible. Coutandin et al. show that the tetramer is considerably more stable than the dimer (top right), and that there is an energy barrier between the two. Phosphorylation triggers the spring-loaded mechanism that enables the system to overcome this barrier (bottom right). The large energy gap between the dimer and tetramer means that the system cannot return to the dimer state.

However, the dimer state cannot simply convert to the lower-energy tetramer state because kinetic effects create an energy barrier that must be overcome to escape from the dimer state. In the spring-loaded model the energy needed to overcome this barrier comes from an external source: in the case of the TAp63a activation this energy comes from phosphorylation. Experiments in which denaturants such as urea were used to mimic the effect of phosphorylation on TAp63 provide support for the spring-loaded activation mechanism.

Some mutant forms of p53 promote the formation of tumors by binding to and altering the activity of other members of the p53 family, including TAp63α (Freed-Pastor and Prives, 2012). Other recent work by the Dötsch lab suggests that a mutant form of p53 that promotes cancer binds to the C-terminal end of other members of the p53 family (Kehrloesser et al., 2016). Coutandin et al. show that when TAp63α forms a dimer, its C-terminal is not accessible, which means that it cannot bind to this mutant form of p53 (Figure 2).

**Figure 2.**
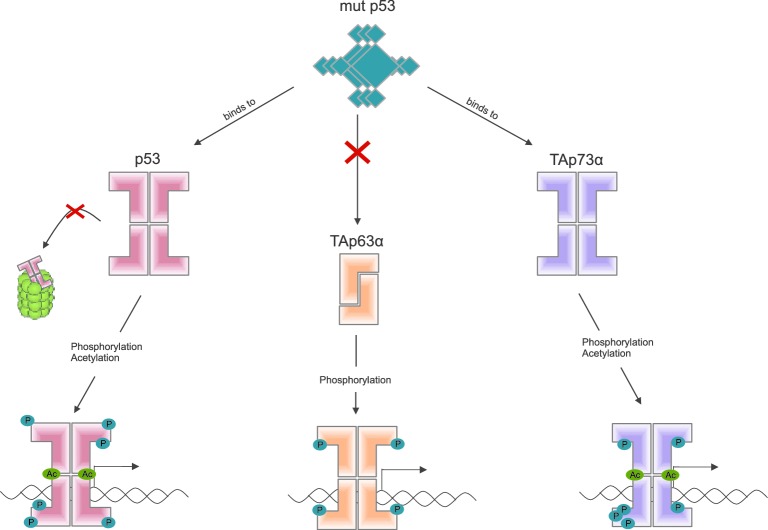
Activation of the different members of the p53 family of transcription factors. TAp63α is a member of the p53 family of proteins, which also includes p53 and p73: these proteins are all transcription factors, so they need to be able to bind to DNA (bottom row). Some mutant forms of p53 can contribute to cancer phenotypes by binding to the C-terminal of normal p53 and p73 proteins: however, these mutant forms cannot bind to TAp63α dimers (because C-terminal regions of the dimers are not accessible). Phosphorylation (blue dots) is the trigger for activation of all three family members, but the mechanism is different for each. For p53 (left) phosphorylation mainly inhibits degradation by proteasomes, thus promoting stabilization of the protein; for TAp73α (right) phosphorylation affects the DNA binding affinity; and for TAp63α (middle) phosphorylation enables the opening up of dimers, which are inactive, in order to form tetramers, which are active. Acetylation (green dots) is also involved in the activation of p53 and TAp73α.

With these recent findings, Dötsch and colleagues continue to expand our knowledge of the link between the biochemical and biophysical properties of the p53 family of proteins and their roles in cells. This improved understanding will help those developing new treatments for female cancer patients who want to have families (conventional chemotherapy can lead to infertility), as well as helping other researchers working on other cancers associated with mutations of p53.
